# The Impact of the Covid-19 Pandemic on Consumers' Intention to Use Shared-Mobility Services in German Cities

**DOI:** 10.3389/fpsyg.2021.646593

**Published:** 2021-03-01

**Authors:** Marion Garaus, Christian Garaus

**Affiliations:** ^1^Department of International Management, Modul University Vienna, Vienna, Austria; ^2^Institute of Marketing and Innovation, University of Natural Resources and Life Sciences, Vienna, Austria

**Keywords:** Covid-19, perceived physical risk, perceived ecological benefits, safety claim, environmental claim, label, mediation analysis, experiment

## Abstract

One sector that severely suffers from the outbreak of the coronavirus is carsharing (i.e., short-term car access). The downswing of the carsharing industry may not only experience negative economic consequences but also ecological ones. Carsharing has the potential to reduce emissions, occupied space, and congestion and hence can actively contribute to mitigating climate change. As Bill Gates strikingly states: “Covid-19 is awful. Climate change could be worse.” For this reason, it is important to understand which underlying mechanisms drive carsharing usage during the Covid-19 pandemic. The current research has the overall objective to provide deeper insights into the mediating mechanisms that explain carsharing usage intention during the Covid-19 pandemic. In particular, we draw on signaling theory to explore how different claims (environmental claims, safety claims) that prompt two different opposing underlying processes (perceived ecological benefits, perceived physical risk) influence carsharing usage intention. An online experiment employing a 3 (environmental claim vs. safety claim vs. no claim) × 2 (high information diagnosticity vs. low information diagnosticity) between-subjects design with participants acquired by the online panel platform Clickworker was conducted in April 2020. Fictitious labels and fictitious advertisements served as stimulus material and constituted the five experimental conditions. The data were analyzed by a multicategorial moderated mediation analysis and a multivariate analysis of covariance. Results reveal that environmental claims can stimulate perceived ecological benefits, which, in turn, positively affect carsharing usage intention. Interestingly, our research demonstrates that safety claims cannot decrease perceived physical risk in the context of Covid-19 and carsharing. Nevertheless, perceived physical risk has a (marginal) negative influence on carsharing usage intention and hence should not be discarded altogether. The findings of this article offer new insights into the mental processes that guide consumer decision-making during the coronavirus crisis and also offer important policy implications by highlighting the relevance of environmental claims during the Covid-19 pandemic. Furthermore, the negative influence of perceived physical risk on carsharing usage intention points to the need for alternative measures to reduce users' risk perceptions.

## Introduction

Within a few months, the global Covid-19 pandemic has changed the mobility patterns of people all over the world. In the U.S., mobility for recreational and retailing purposes decreased by −35% and visits to workplaces dropped by −37% in April 2020. In Germany, the reduction of mobility behavior was even severer for recreational and retailing purposes (−51%); for workplace mobility, the drop was −38% (Google, 2020). When it comes to mobility choices, the Covid-19 pandemic seems to have caused a shift in priorities regarding mobility too. Before the crisis, travel time, the cost or price of the service, and the convenience of the trip as well as different socio-economic variables unique to the individual have been identified as major criteria affecting decisions about modes of transport (Muro-Rodríguez et al., 2017). However, during the crisis, the reduced risk of infection may have replaced the time factor as the highest priority (Andersson et al., 2020). Among others, it is likely that these health considerations resulted not only in a significant decrease in the use of public transport (U.S. −31%, Germany: −48% visits of transit stations in April 2020; Google, 2020) but also a decrease in carsharing usage since then, especially in markets where carsharing has frequently been used. For instance, Italy noted a decrease in the use of carsharing services by 60% with peaks as high as 70% (Deloitte Italy, 2020). While the pandemic seems to have caused a decrease in usage among all mobility services, a survey with 1,000 respondents in Germany in April 2020 revealed that 70% of participants used a private car to commute to work (conducted by the German Aerospace Center, 2020a). In a follow-up study in August 2020, the researchers confirmed that the private car is the clear winner in the Covid-19 pandemic (German Aerospace Center, 2020b). When compared to private car use in Germany before the Covid-19 pandemic, the results reveal an increase of 16% during the first lockdown compared to an increase of 5% after the lockdown. In confirmation of these findings, 32% of all households without a car indicated that they missed having their own car during the lockdown though this figure decreased to 19% after the lockdown, which still constitutes a substantial amount (German Aerospace Center, 2020c). Individuals reported feeling uncomfortable when using public transport or carsharing services, which comes at the cost of increased use of private cars (German Aerospace Center, 2020b). Although some countries offer guidance on how to minimize the risk of a Covid-19 infection in vehicles provided by commercial operators (Transport Canada, 2020), the lack of obligations when it comes to cleanliness and protective equipment seems to have raised doubts about the safety of carsharing services. Indeed, the most prominent carsharing provider in Germany, ShareNow, reported a drop in carsharing usage by 56% in Hamburg and 62% in Berlin in April 2020 as compared to the same period of the previous year. Additionally, following the lockdown, this decrease persisted albeit at a lower level (11% in Hamburg and 7% in Berlin).^1^

Consequently, carsharing service offers were reduced or, in some cases, services were even suspended during lockdowns (Bert et al., 2020). Despite carsharing providers' efforts to communicate safety measures (e.g., ShareNow, 2020a), a recent survey reveals that only five to eight percent of 8,000 surveyed customers think that shared-mobility services are safe from a health perspective (Andersson et al., 2020). The Covid-19 pandemic makes consumers withdraw from services used before the pandemic and seek products and services associated with high levels of safety (Blazquez-Resino et al., 2020).

While the Covid-19 pandemic negatively affects almost all sectors of the global economy (El Keshky et al., 2020), the suffering of the carsharing economy must be considered from an environmental perspective as well. In 2016, a survey in five North American cities revealed that the provision of carsharing services has the potential to reduce private car acquisition while increasing private vehicle selling (Martin and Shaheen, 2016). Carsharing has been found to reduce car ownership, car kilometers, and ultimately reduces CO_2_ emissions (Nijland and van Meerkerk, 2017). In particular, when carsharing substitutes car ownership or when the carsharing provider makes use of electrical cars, carsharing can have significant positive environmental impacts (Wappelhorst et al., 2014; Firnkorn and Müller, 2015). While climate change is by no doubt an important topic, recent figures emphasize once more its relevance: Climate change and the associated rise in temperatures around the globe could cause more annual deaths than infectious disease in 2100 (Gates, 2020; Worland, 2020). The causes of these increasing death rates can be ascribed to heat stress, malnutrition, malaria, and diarrhea (Lyons, 2019). Hence, the drop in public transport and carsharing services, including the associated preference for private car use, both during and after the Covid-19 pandemic (German Aerospace Center, 2020a) results not only in negative economic impacts but may also lead to detrimental environmental impacts. This holds especially when consumers make long-term decisions that come at the cost of carsharing [e.g., the purchase of an (additional) private car].

The economic and environmental impacts of declining carsharing usage call for a multifaceted perspective on the underlying mechanisms that drive carsharing usage during the coronavirus crisis. Research is called for that helps to understand cognitive processes when making consumption-related decisions during and after the Covid-19 pandemic (Blazquez-Resino et al., 2020). The complexity of consumer behavior in this context requires the consideration of opposed mechanisms that drive individuals to make use of carsharing services. More specifically, we postulate that the perception of ecological benefits is driving carsharing usage. While there is a growing interest in sustainable products and services (Atkinson and Rosenthal, 2014), environmental benefits have been identified as a nice add-on rather than a driver of carsharing usage (e.g., Lindloff et al., 2014; Hartl et al., 2018; Ramos et al., 2020). Our research offers new important insights into the role of perceived ecological benefits by employing causal research. More specifically, our research reveals that perceived ecological benefits serve as an underlying mechanism that explains carsharing usage also during the Covid-19 pandemic. Hence, the findings of our experiment emphasize the relevance of clearly stating ecological benefits when offering carsharing services. At the same time, perceived physical risk must be considered as an important process that stands in the way of carsharing-usage decisions during the coronavirus crisis. The physical risk dimension in the construct of perceived risk has not attracted much research attention in the past since most products are more associated with other kinds of risk, such as financial risk or time risk (see Stone and Grønhaug, 1993). Also, extant literature in the context of carsharing focuses on comparing the financial, performance, and social risks of carsharing usage to those of car ownership (Schaefers et al., 2016). Nevertheless, with the rise of the Covid-19 pandemic, the physical risk dimension has become the dominant one. Users are concerned about getting infected and perceive an unprecedented level of physical risk (Andersson et al., 2020), which opposes the positive effects of the perceived ecological benefits of carsharing. By drawing on signaling theory as an underlying framework, we explain the opposing processes of perceived environmental benefits and perceived risk on the influence of different labels on carsharing usage.

## Conceptual Research Framework

### Carsharing

Carsharing is commonly considered to be one of the most important sectors of the sharing economy, driving its growth from 15 bn in 2014 to a projected revenue of USD 335 bn in 2025 (Hawksworth and Vaughan, 2014). As for any other sector of the sharing economy, the intention is to replace the ownership of an underused asset by sharing it with peers (Botsman and Rogers, 2010). Carsharing is provided as a shared-mobility service that allows users to share vehicles of a fleet (Nourinejad and Roorda, 2015). How this “mobility as a service” is organized can be categorized into two dominant forms: station-based and free-floating carsharing. Station-based carsharing, which already emerged in the late 1980s and may be operated by cooperative, non-profit, or for-profit providers, is typically a two-way system that requires a customer to pick up and return a car from a fixed spot (Nourinejad and Roorda, 2015; Vaskelainen and Münzel, 2018). Free-floating carsharing allows customers to temporarily use a car and leave it at the trip destination, where the next user will pick it up. This one-way system of carsharing emerged in the late 2000s at a time when costs for the required coordination and communication among users and service operators sharply dropped due to the rise of internet-based platform technologies (Matzler et al., 2015; Vaskelainen and Münzel, 2018). Despite its higher complexity and the associated challenges of free-floating carsharing as compared to station-based carsharing services (Terrien et al., 2016), free-floating carsharing provides more flexibility and lower costs for the driver and is likely to grow in the next decade, especially in metropolitan areas (Shaheen et al., 2015).

Free-floating carsharing, which is typically operated by a for-profit provider, needs a “critical mass” of users to operate. The area of service of free-floating carsharing is thus typically limited to a city, currently mostly in Europe (more than 50% of the global carsharing market) and North America with ShareNow leading the global market (Deloitte Germany, 2017). However, the rapid growth of the carsharing sector is not entirely driven by technology; changes in the consumption patterns of customers also play an important role (Shaheen et al., 2020).

While ownership has often been considered to be the most desirable way to access cars (Matzler et al., 2015), users are increasingly turning to “access-based consumption.” Phenomenological research before the Covid-19 pandemic has tried to uncover the reasons why individuals use carsharing services. Schaefers' (2013) qualitative study of carsharing users found four motives for using carsharing: value seeking (e.g., spend less than to own a car, free parking), convenience (e.g., easily find parking spots, fewer responsibilities), lifestyle (e.g., something to talk about, recognized by others), and environmental motives (e.g., fuel efficiency, ability to go carless). Several studies have found that value seeking and convenience are the main drivers as to why individuals use carsharing services while environmental motives are considered to be secondary or a positive side effect (e.g., Lindloff et al., 2014; Hartl et al., 2018; Ramos et al., 2020). Also, a recent quantitative study by Münzel et al. (2019) confirms these findings, but highlights—similar to Efthymiou et al. (2013) before—that environmental motives positively influence the usage intention of carsharing and call for more research to deepen the understanding of motives for carsharing on the individual level.

On an aggregated, system-wide level, environmental benefits are often the focus of research, policy-making, and management practice as carsharing models are widely considered to be a sustainable mode of transportation (Kortum et al., 2016). Prior research demonstrates that carsharing holds the potential to reduce the number of car kilometers driven, to increase the offer of electric or less pollutant cars, to incentivize modal shifts toward other sustainable modes of transport (e.g., public transport, walking, and cycling), and to reduce the number of cars owned (cf. Münzel et al., 2019). Jochem et al.'s (2020) survey-based study on the impact of carsharing on private car ownership across 11 European cities revealed that one free-floating carsharing service replaces at least eight private cars and, in optimistic scenarios, even up to 20 cars. In this context, shared mobility services in general and free-floating carsharing in specific can mitigate public costs associated with personal car use and help cities in their development of a sustainable transportation strategy (Terrien et al., 2016; Shaheen et al., 2020).

Accordingly, an increase in carsharing opportunities in a city might contribute to a better climate and might be a promising measure to mitigate climate change. Unfortunately, the ongoing coronavirus crisis hurts the recorded trend of carsharing usage to date. Not only reduced mobility caused by lockdowns all over the world but also the challenge to meet safety requirements in shared cars challenges the industry. Indeed, most of the potential users of carsharing services that were surveyed indicate major concerns regarding the safety of carsharing during the Covid-19 pandemic (Andersson et al., 2020). In confirming the validity of these concerns, research reports that the virus remains on plastic and stainless steel, which are materials often utilized in cars, for hours or even days. Goldman (2020), for instance, proposes that a risk of infection exists if a person touches a surface immediately after (1–2 h) an infected person directly coughed or sneezed on it. Other scholars suggest that the virus may survive between 2 or 3 days on dry surfaces (Van Doremalen et al., 2020) or even up to 9 days at room temperature (Akram, 2020; Fiorillo et al., 2020). Hence, touching surfaces while traveling carries the risk of infection with the coronavirus, with the original transmitter being unknown and hard to trace for those that are infected (Suman et al., 2020). Other compelling evidence reports that a higher frequency of Covid-19 cases is associated with increased use of public transport for similar reasons (Loomba et al., 2021).

In response to these concerns, carsharing providers have started to inform users of the specific measures taken to assure the safety of the user of the service. For instance, the carsharing provider ShareNow offers detailed information on the benefits of using carsharing services instead of other modes of transport during the pandemic as well as hygiene measures and advice on how to reduce the risk of infection (ShareNow, 2020a). Based on signaling theory, such an approach might be a promising way to communicate certain benefits of products and services.

### Signaling Theory

Signaling theory explains how cues can be used to influence consumers' perceptions. The signaling theory has been developed with the example of job markets (Spence, 1973). Recruiters' decisions on hiring new employees are associated with high levels of uncertainty since not all applicants' capabilities are visible at the time of making hiring decisions. For this reason, applicants invest in various signals (e.g., education) to signal superior value as compared to competing applicants (Spence, 1973). Since its introduction in 1973, signaling theory has received considerable attention by scholars studying it in manifold management and marketing contexts. Especially when asymmetry between two actors in a business transaction is high, managers are well-advised to use signals to communicate the quality of products (Mishra et al., 1998). In the context of marketing, labels are often used to signal specific product benefits.

### Environmental Claims and Perceived Ecological Benefits

Environmental claims have been identified as an effective strategy to inform consumers that a given company employs tactics and processes to protect the environment (Gutierrez et al., 2020). Eco-labels serve as certifications to assure consumers of the quality of a product or service (Atkinson and Rosenthal, 2014). In the context of tourism, research reveals that carbon labels (i.e., labels providing information on CO_2_ emissions) can contribute to more sustainable choices (Gössling and Buckley, 2016). Well-designed eco-labels influence consumers' eco-friendliness perceptions of the product or service in question (Teisl et al., 2008). Research in the context of cars demonstrates that eco-labels have the potential to overcome information asymmetry between consumers and sellers (Codagnone et al., 2016). Following this line of reasoning, we propose that:

H1. An environmental claim increases the perceived ecological benefits of carsharing.

In the context of retailing, research demonstrates that consumers often base their purchase decisions on product labels (Berry et al., 2015). Environmental claims help consumers to make sustainable purchase decisions (Gutierrez et al., 2020). In general, studies report that consumers prefer environmentally-friendly products and are willing to pay a price premium for eco-labeled products (Loureiro and Lotade, 2005). The perception of ecological benefits (e.g., reduced emissions) positively influences consumers' purchase intentions (Hartmann and Apaolaza-Ibáñez, 2012). In support of this finding, another study reports that positioning a product as “green” positively influences purchase intentions (Mohd, 2016). However, signals influence consumer behavior only when consumers experience them as useful and credible (Boulding and Kirmani, 1993). Extant research emphasizes that—for the efficiency of environmental labels—consumers need to understand the information conveyed by the label and experience the conveyed information as significant (Gössling and Buckley, 2016). A recent study reveals that perceived benefits serve as an important predictor of carsharing usage intention (Acheampong and Siiba, 2020). Hence, we assume that environmental claims lead to perceived ecological benefits and that perceived ecological benefits serve as an underlying mechanism explaining how eco-labels influence usage intention of carsharing services. We suggest that:

H2. Perceived ecological benefits increase carsharing usage intention.
H3. Perceived ecological benefits mediate the influence of an environmental claim on carsharing usage intention.

### Safety Claims and Perceived Physical Risk

Claims assuring that product usage is safe have received considerable research attention in the context of food. For instance, front-of-package labeling is an effective means to help consumers to identify healthier products (Ikonen et al., 2020). However, recently, practical evidence suggests that safety claims are often used to assure consumers that the use of a given service is Covid-19 safe. In the last months, several health claims have emerged that relate to Covid-19 hygiene measures. For instance, the HRS hotel group promotes its lodging services by using a “Clean & Safe” label (HRS, 2020). Likewise, the European city of Brussels launched a health and safety label to stimulate tourism by assuring tourists of the quality and safety of the city's infrastructure, for instance, by using chemical disinfectants in public transport (Visit Brussels, 2020). Indeed, research reveals that the used chemicals 0.1% sodium hypochlorite or 62–71% ethanol effectively disinfects surfaces when applied for 1 min and thus confirms the effectiveness of employing increased hygiene measures to reduce the spread of the coronavirus (Fiorillo et al., 2020). The theoretical reasoning for using such labels can be found in perceived risk literature. In the context of consumer behavior, perceived risk defines the uncertainty of potential consequences, of which some might be unpleasant, involved in every purchase decision (Bauer, 1960). One important dimension of perceived risk represents the physical risk (Jacoby and Kaplan, 1972). During the Covid-19 pandemic, physical risk in the context of carsharing reflects the possibility that the coronavirus might spread in the car and hence might injure the consumers' health.

Consumers tend to manage their risk in purchase situations (Mitra et al., 1999). Perceived risk is higher if a product or a service is associated with high levels of uncertainty, which is usually the case if consumers lack information to assess the service or product quality in advance (Taylor, 1974). Credence attributes (i.e., service or product characteristics that cannot be confidently evaluated by consumers before, during, or after the purchase) are associated with high levels of perceived risk as compared to other attributes (Mitra et al., 1999). However, the provision of information can diminish risk perceptions as a study in the context of food safety demonstrates (Yeung et al., 2010). A quality assurance label can reduce consumers' perceived risk in the context of microbiological risk in chicken meat (Yeung et al., 2010) as well as for using new technologies (Douthitt, 1995). Following this line of thinking, we propose that a safety claim communicating that the offered service is Covid-19 safe can diminish consumers' physical risk perceptions in the context of carsharing. More formally, we hypothesize that:

H4. A safety claim decreases the perceived physical risk of carsharing.

Perceived risk is an important construct in marketing since it is negatively correlated with purchase intentions (Yeung et al., 2010), which is in turn an important predictor of actual usage behavior (Venkatesh et al., 2012). Perceived risk negatively impacts consumers' usage intentions of mobile payment services (Thakur and Srivastava, 2014). In another online shopping context, research confirms that lower levels of perceived risk lead to higher purchase intentions of apparel (Park et al., 2005). Indeed, perceived risk is often used to determine preferences among many product alternatives. However, if a purchase decision is associated with a high level of risk, consumers might not purchase at all (Schaefers et al., 2016). In a similar vein, we argue that if consumers experience high levels of physical risk with the use of carsharing services, consumers will have lower usage intentions. Furthermore, we argue that the diminishing effect on perceived risk from health labels affects purchase intention. Accordingly, we suggest that:

H5. Perceived physical risk negatively influences carsharing usage intention.
H6. Perceived physical risk mediates the influence of a safety claim on carsharing usage intention.

### Environmental Claims Increase Physical Risk Perceptions

While well-designed and informative eco-labels likely increase ecological benefits, it is also reasonable to assume that they backfire when it comes to risk perception. An eco-label might stimulate consumers' thoughts if it is used to mask more pressing issues during the Covid-19 pandemic, namely, safety aspects. During the Covid-19 pandemic, consumers most likely expect a label indicating that the use of the specific service is Covid-19 safe. However, if an ecological label is provided instead of the traditional safety claim, consumers might draw the wrong conclusion that the carsharing provider cannot meet safety guidelines. To compensate for the lack of safety, another benefit, namely, an ecological benefit, is communicated. Indeed, existing research in related contexts indicates that consumers often draw the wrong conclusion based on labels. For instance, consumers tend to eat more unhealthy products when nutrition information as cues does not meet consumers' calorie expectations (i.e., when the calorie level is lower than expected). Other studies have demonstrated that environmental claims fail to prompt any health inferences for food products (Larceneux et al., 2012). Likewise, research reveals that “no cholesterol”-labels decrease consumers' fat perceptions (Andrews et al., 1998). In line with this argumentation, we likewise propose that consumers might draw the wrong conclusion from an environmental claim for risk perceptions. Our hypothesis reads:

H7. An environmental claim increases the perceived physical risk of carsharing.

### Moderating Influence of Information Diagnosticity

One strategy to convince consumers of the usefulness and credibility of labels is their specificity. For instance, labels with very vague claims, such as “natural” or “healthy,” do not contain specific information and hence elicit weaker consumer responses as compared to specific claims, which include specific attributes on how the label assures the sustainability of a given product or service (Atkinson and Rosenthal, 2014). To make an informed purchase decision, consumers require information that helps them to differentiate between various market offerings (Menon et al., 1995). In the context of environmental claims, vague statements might irritate consumers (Orazi and Chan, 2018). Transferring this reasoning to the context of environmental and safety claims for carsharing providers implies that more detailed information strengthens the positive influence of environmental claims on perceived ecological benefits as well as the positive impact of safety claims on perceived physical risk. We hypothesize that:

H8. Higher information diagnosticity positively influences the impact of (a) an environmental claim on perceived ecological benefits and (b) a safety claim on perceived physical risk.

## Materials and Methods

### Subjects

Participants for our experiment were recruited from the online panel provider Clickworker, which uses a self-selection sampling method (cf. Colman, 2015). Clickworker enables the online recruitment of survey participants by issuing an open call to participate in a study (Clickworker, 2021). To qualify for our study, panelists needed to be fluent in German (roughly 12% or 260,000 members of the Clickworker crowd) and were required to fulfill the criteria of (1) owning a driver's license, (2) having the center of life in Munich, Berlin, Frankfurt, Stuttgart, Düsseldorf, Cologne, or Hamburg (since the carsharing provider used for the stimulus, ShareNow, is available in these cities), and (3) having a general interest in carsharing. This selection process resulted in 240 qualified participants (42% female), aged between 18 and 61 (mean age 36). About two thirds of the participants held at least a high school degree; more than half (54%) held a university degree. Thus, the sample is similar to the population of ShareNow users, who tend to be male, between 25 and 45 years old, live in densely populated urban areas, and have higher educational qualifications (Kopp et al., 2015).

### Study Design, Stimulus, and Procedure

**Figure 1:**
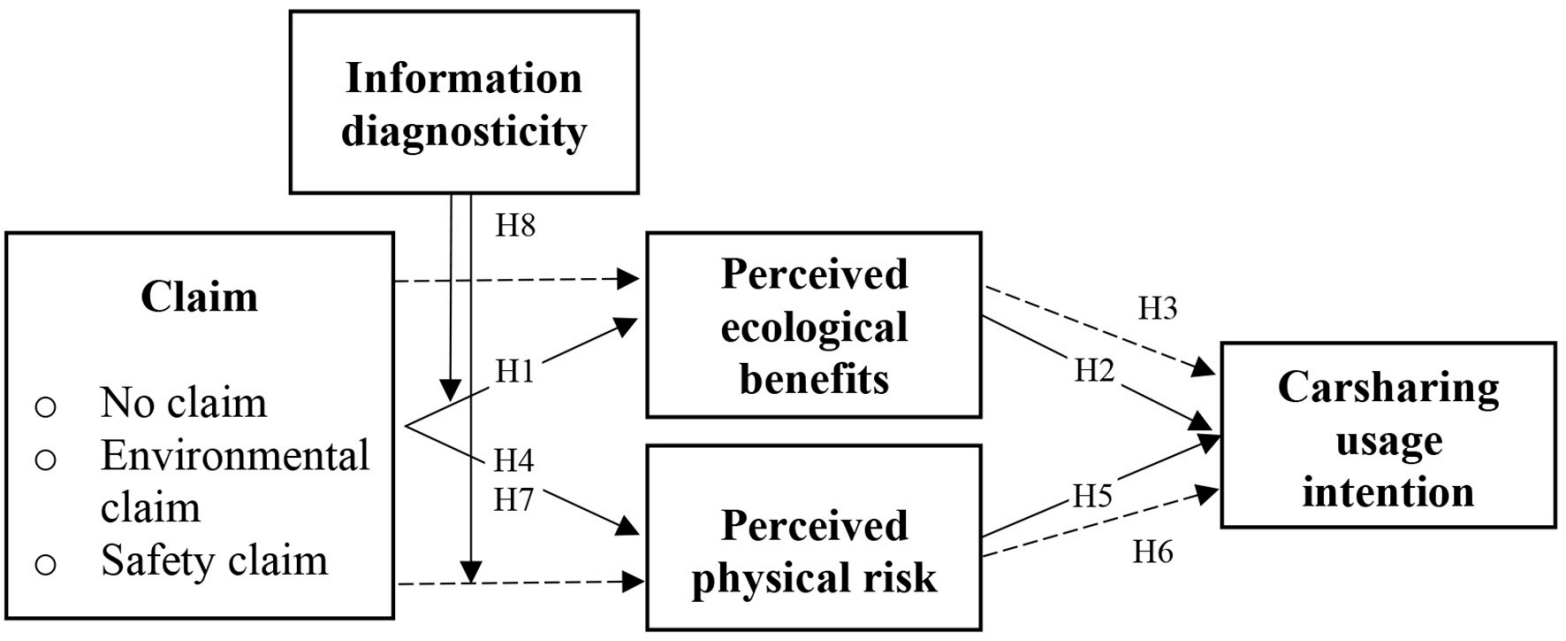
Conceptual research framework.

**Figure 2:**
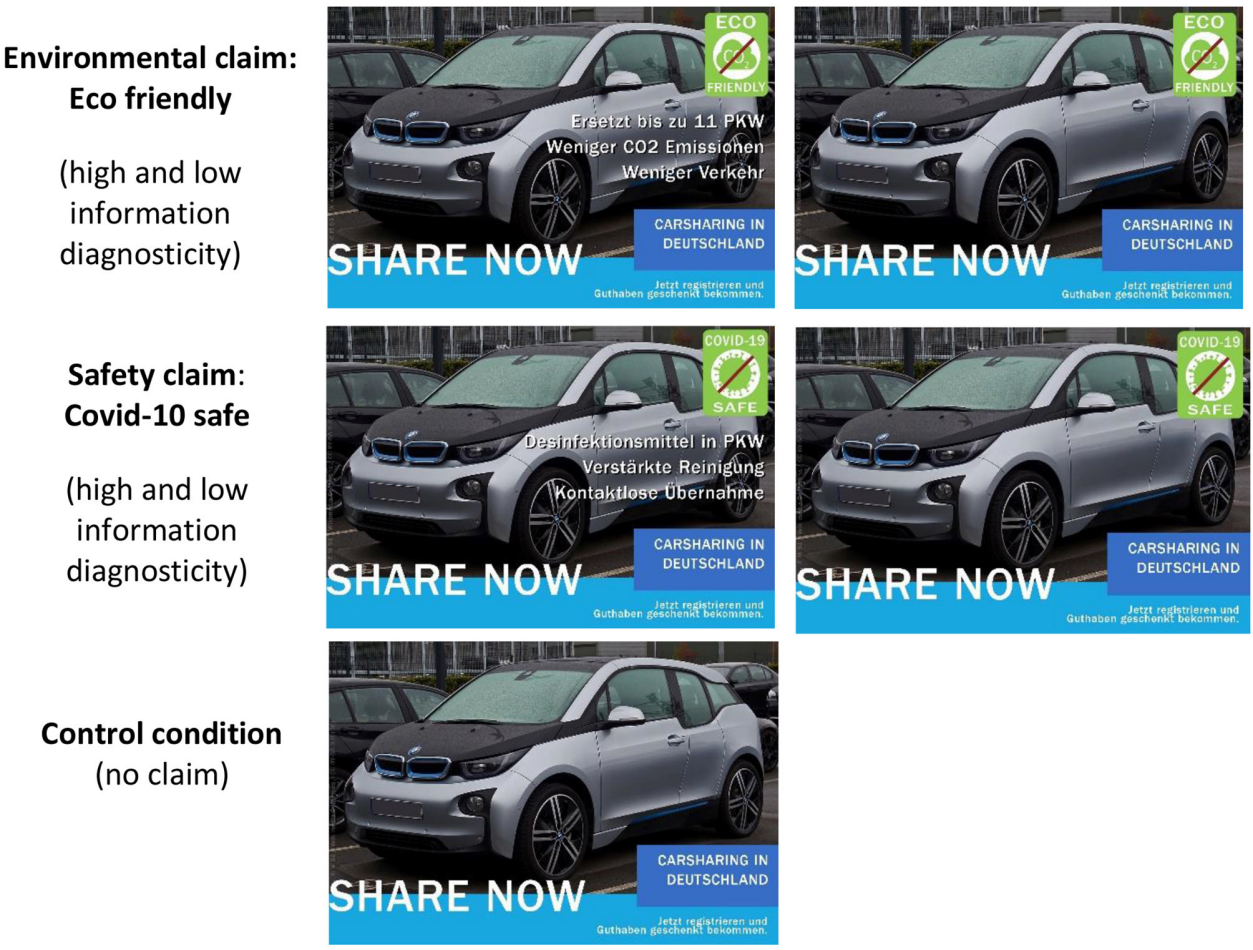
Manipulation of experimental conditions (environmental claim vs. safety claim vs. control condition).

An online experiment was conducted in October 2020 to test the conceptual research framework and the associated hypotheses (see Figure 1 for an overview). A fictitious advertisement based on a picture from the website Wikipedia Commons (2014) promoting a well-known carsharing service—ShareNow, the world's largest provider of free-floating carsharing—was used as stimulus material. ShareNow (and car2go and DriveNow, the precursory organizations before their merger in 2019) have been frequently investigated in prior studies (e.g., Firnkorn, 2012; Kortum et al., 2016; Jochem et al., 2020). Our stimulus focused on the German market, which is ShareNow's largest one (ShareNow, 2020b). The environmental claim was manipulated via the presence or absence of an eco-label (cf. Gutierrez et al., 2020) located in the upper right corner of the advertisement. Likewise, a Covid-19 safe label was used to manipulate a safety claim. Furthermore, information diagnosticity was manipulated by adding three bullet points, providing consumers with more detailed information (Atkinson and Rosenthal, 2014) on environmental claims (replaces up to 11 cars, less CO_2_ emissions, less traffic) or safety claims (disinfectants in cars, reinforced cleaning, contactless takeover). In addition, a control condition without any claim was created. Hence, a two-factor (environmental claim vs. safety claim × high information diagnosticity vs. low information diagnosticity + control condition) between-subject design was employed (see Figure 2).

Participants were randomly allocated to one of the five experimental groups. The questionnaire started with a short introduction about the purpose of the study, namely, carsharing usage behavior. After qualifying for the study by positively answering the questions related to a driver's license, center of life, and interest in carsharing, participants were exposed to one of the five fictitious advertisements for at least 20 s. Afterwards, the questionnaire began with items assessing the success of the manipulation, the mediating constructs, and the dependent variable. The questionnaire ended with the assessment of demographic variables as well as prior carsharing usage.

### Measures

To assess whether the manipulation worked successfully, one item asked respondents to indicate whether the advertisement included a label and, if so, which kind of label. All scales assessing the mediating constructs and the dependent variable were measured on established scales (if not indicated otherwise, all constructs were measured on a Likert Scale anchored from 1-strongly disagree to 7-strongly agree) Three items assessed information diagnosticity (Hernandez et al., 2014). Perceived ecological benefits were measured by three items adapted from Acheampong and Siiba (2020). The perceived physical risk was assessed by items derived from the research of Jacoby and Kaplan (1972). Three items adopted from Thakur and Srivastava (2014) evaluated participants' intention to use the promoted carsharing service. As control variables, we assessed the extent to which respondents perceived the advertisement as appealing and the familiarity with the promoted carsharing service (Simonin and Ruth, 1998). We further controlled for age, gender (female vs. male), education (with secondary education vs. without secondary education), advertising appeal, prior usage of the ShareNow carsharing provider in the past year (yes vs. no). Finally, one item measured the participants' usual mode of travel (Acheampong and Siiba, 2020). Table 1 summarizes the items constituting the various constructs used in the questionnaire and construct reliabilities (see Table 1).

**Table 1:** Measurement of constructs.

**Construct/items**	**Cronbach's alpha**
**Manipulation check (label)** Did you see a label (claim) in the advertisement? Please tick whether you have seen a label and, if so, which one (answer options: Yes, an ECO label; Yes, a Covid-19 label, No).	
**Information diagnosticity** (Hernandez et al., 2014) The information in the advertisement was sufficient to evaluate this carsharing service. The advertisement gives detailed information about the advantages of this carsharing service. The special features of this carsharing service are explained in detail.	0.88
**Perceived physical risk** (Jacoby and Kaplan, 1972) Please indicate to what extent you agree with the following statements *7-point Likert scale (strongly disagree—strongly agree)* In the current situation… … using this carsharing service could endanger my health. … using this carsharing service would be harmful to health. … it would not be safe to use this carsharing service.	0.89
**Perceived ecological benefits** (Acheampong and Siiba, 2020) Please indicate to what extent you agree with the following statements *7-point Likert scale (strongly disagree—strongly agree)* In the current situation… … using this carsharing service could reduce traffic congestion. … using this carsharing service could reduce pollution. … using this carsharing service could reduce the ownership of cars.	0.66
**Carsharing usage intention** (Thakur and Srivastava, 2014) Please indicate to what extent you agree with the following statements *7-point Likert scale (strongly disagree—strongly agree)* I am considering using this carsharing service in the future. I assume that I will use this carsharing service in the future. It is likely that I will use this carsharing service in the future.	0.91
**Familiarity** (Simonin and Ruth, 1998) Please indicate to what extent you know the carsharing provider “ShareNow” *Semantic differential (−3 to +3)* Unfamiliar/Familiar Not recognized/Recognized Had not heard about/Had heard about	0.89
**Travel mode** (Acheampong and Siiba, 2020) What is your usual way of getting around? *Single choice* Private-car or motorcycle (driving alone) Private-car (car-pooling) Public transport Non-motorized (walking/cycling)	

### Preliminary Data Analysis and Testing Assumptions

The random assignment of participants to one of the experimental groups ensured that the data meets the assumption of independence of observations. The assumption of normality distribution for the two mediating constructs of perceived physical risk and perceived ecological benefits as well as for the dependent variable of carsharing usage intention was tested following the procedure suggested by Field (2009): In a first step, a visual inspection of the histograms offers the first indication that our mediating and dependent variables are nearly normally distributed as they are bell-shaped and resemble the normal distribution. In a second step, the *z*-scores of the kurtosis and skewness are calculated. As a normal distribution has a kurtosis and a skewness of zero, values that are close to zero suggest that the distribution is nearly normal. While the *z*-scores of the skewness of perceived physical risk (1.62) and the skewness of perceived ecological benefits (−2.97) was satisfactory, the *z*-score of kurtosis of usage intention (−3.42) slightly exceeded the upper threshold of 3.29. However, the kurtosis *z*-score of usage intention (−0.06) is not of any concern nor is the kurtosis *z*-scores of perceived physical risk (−2.77) nor perceived ecological benefits (1.31). In addition, we expect robustness of our results because of the central limit theorem. Pek et al. (2018) emphasize that the assumption of normality can be relaxed when the sample size is large enough; in their paper, they judge a sample size of roughly 100 as sufficiently large. Field (2009) similarly highlights that studies with sample sizes larger than 200 should rely on the visual inspection only, leading us to conclude that the assumption of normality is inessential with this study's sample size of 240 (Pek et al., 2018). For the main analysis, we additionally utilize bootstrapping, alleviating a potential concern about non-normality even further (Hayes, 2017).

Scatterplots for the variables of perceived physical risk, perceived ecological benefits, and usage intention as well as the variables for the covariates of advertising appeal and familiarity confirm that our data meets the assumption of a linear relationship between each pair of independent variables, each pair of dependent variables, and each pair of both independent and dependent variables and covariates. The homogeneity of regression slopes among the experimental conditions claim and information diagnosticity were assessed by specifying interaction effects between the experimental conditions and the six covariates. The analysis reveals non-significant interaction effects among all tested interaction effects: claim × age: Pillai's trace = 0.00, *F*_(4, 430)_ = 0.24, *p* = 0.92, claim × gender: Pillais's trace = 0.02, *F*_(4, 430)_ = 1.19, *p* = 0.31, claim × education: Pillais's trace = 0.01, *F*_(4, 430)_ = 0.69, *p* = 0.60, claim × familiarity: Pillai's trace = 0.02, *F*_(4, 430)_ = 0.90, *p* = 0.46, claim × ad appeal: Pillai's trace = 0.00, *F*_(4, 430)_ = 0.15, *p* = 0.96, claim × ShareNow: Pillai's trace = 0.03, *F*_(4, 430)_ = 1.36, *p* = 0.25, information diagnosticity × age: Pillai's trace = 0.01, *F*_(2, 214)_ = 0.88, *p* = 0.42, information diagnosticity × gender: Pillai's trace = 0.02, *F*_(2, 214)_ = 2.36, *p* = 0.10, information diagnosticity × education: Pillai's trace = 0.01, *F*_(2, 214)_ = 0.86, *p* = 0.42, information diagnosticity × familiarity: Pillai's trace = 0.00, *F*_(2, 214)_ = 0.02, *p* = 0.98, information diagnosticity × advertising appeal: Pillai's trace = 0.01, *F*_(2, 214)_ = 1.57, *p* = 0.21, information diagnosticity × ShareNow. The information diagnosticity × ShareNow interaction represents an exception and reveals a significant result: Pillai's trace = 0.03, *F*_(2, 214)_ = 3.45, *p* = 0.03. Hence, any potential significant interaction effect between these two variables has to be interpreted with caution.

Following the suggestion of Tabachnick and Fidell (2000), we used a Box's M test to check for equal sample sizes among all conditions (the maximum difference between all conditions was nine participants with an average of 50 observations per group). The test was performed by a MANCOVA analysis, using the experimental conditions label and information diagnosticity as factor variables: perceived ecological benefits, perceived physical risk, and all covariates used in the main analysis (familiarity, age, gender, education, advertising appeal, and prior usage of ShareNow carsharing services) were used as covariates. The test revealed a non-significant result (*p* = 0.12). Hence, the covariance matrices can be considered equal and the assumption of homogeneity to be met. An inspection of boxplots for all three variables (perceived physical risk, perceived ecological benefits, and usage intention) among the three label conditions reveals the absence of any outliers. Finally, the absolute values of the correlation coefficients among the study variables were all below 0.42, indicating the absence of multicollinearity.

### Analysis and Results

To test if there is a relationship between the experimental manipulation of the claim (safety claim vs. environmental claim vs. no claim), a Pearson's chi-square test was conducted with the two categorial variables environmental condition and the manipulation check measure (“have seen a label and, if so, which one?”). The result revealed that participants were able to differentiate between the different claim conditions (i.e., environmental claim vs. safety claim vs. no label) [$\chi_{\left(4,\;\text{N}=240\right)}^{2}$ = 294.05, *p* < 0.01]. In a subsequent step, the success of the manipulation of information diagnosticity was tested by an ANOVA, using the information diagnosticity experimental condition (low vs. high) as the factor variable and the mean score of the three items for assessing information diagnosticity (Hernandez et al., 2014) as the dependent variable. The analysis demonstrated that participants evaluated the advertisement with additional information as higher in information diagnosticity as compared to the advertisements that contained only a label without any additional information [*F*_(1, 188)_ = 14.06, *p* = 0.01, M_low_ = 3.52, SD = 1.53 vs. M_high_ = 4.30, SD = 1.26]. Hence, the manipulation of the two factors, claim (environmental claim vs. safety claim vs. no claim) and information diagnosticity (high information diagnosticity vs. low information diagnosticity) worked as intended.

The conceptual model was tested with a multi-categorical mediation model (model 4, 10,000 bootstrap samples, 95% CI) PROCESS v3 macro for SPSS (Hayes, 2017). The multi-categorial mediation model requires dummy coding for the experimental conditions, wherein the control group (no claim condition) serves as the reference category. Hence, the environmental-claim condition and the safety-claim condition are compared to the no-claim condition. In contrast to mediation analysis with dichotomous or continuous variables, this implies that we do not obtain only one direct and one indirect effect (Hayes, 2017). Due to the multicategorial nature of the independent variable, the set of regressions coefficients increases, leading to the estimation of four relative direct effects (*a*_1_ –* a*_4_*)* on the two mediating constructs of perceived ecological benefits and perceived physical risk and two relative direct effects on usage intention ($c_{1}^{\prime },c_{2}^{\prime }$) from the independent variable (i.e., the experimental conditions). The no-claim condition served as a reference category, so all effects must be interpreted in comparison to the no-claim condition.

**Figure 3:**
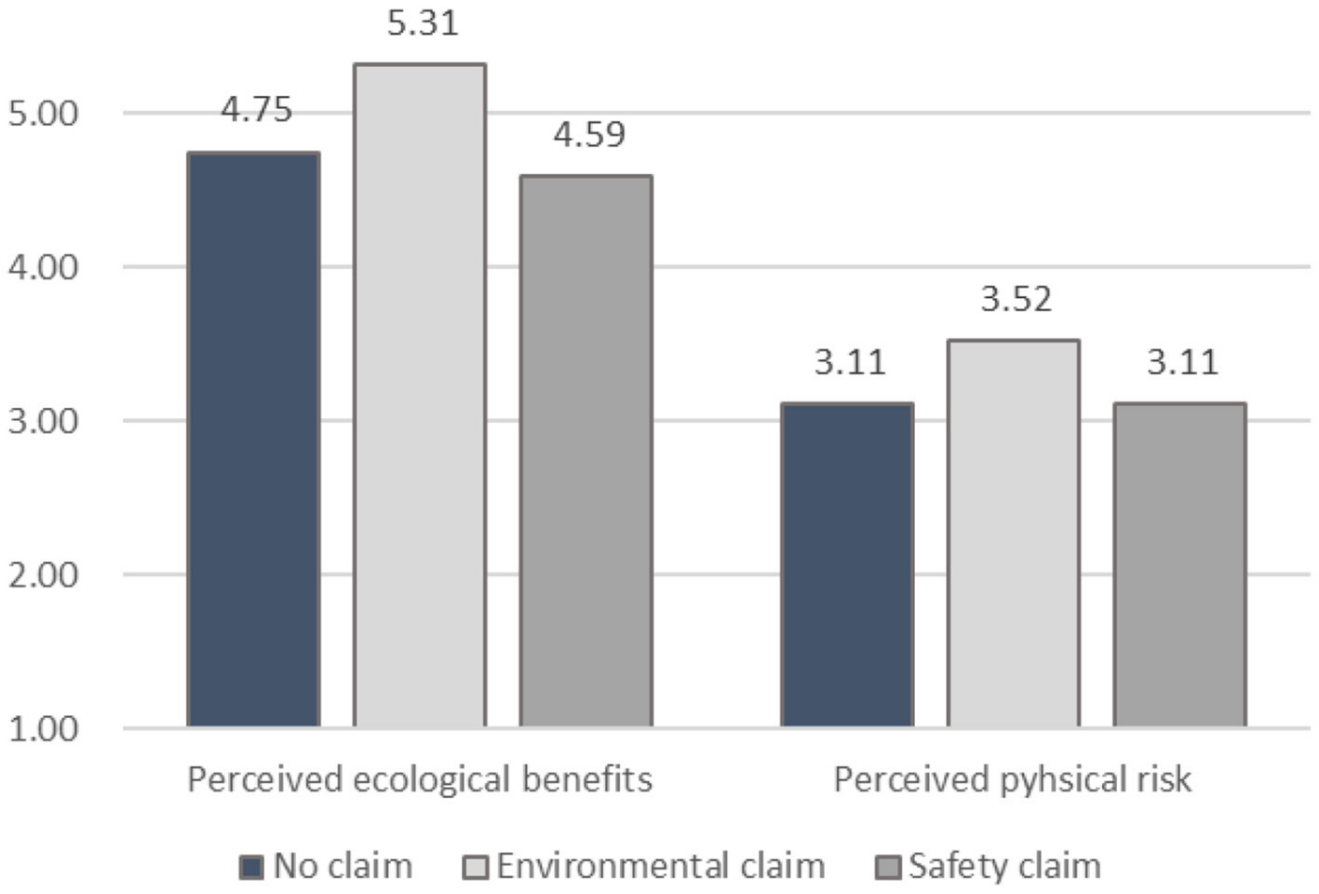
Mean comparisons among the three experimental conditions.

The inspection of the direct effects (*a*_1_*, a*_3_) from the experimental condition of environmental-claim on perceived ecological benefits and perceived physical risk confirms H1 and H7 (see Table 2). The environmental claim had a significant positive effect on perceived ecological benefits (*a*_1_= 0.46, *p* = 0.03) and significantly increased perceived physical risk (*a*_3_= 0.58, *p* = 0.04) when compared to the no-claim condition. In corroboration of H2, the direct effect of perceived ecological benefits on carsharing usage intention was significant (*b*_1_= 0.30, *p* < 0.01). An inspection of the indirect effect (environmental claim → ecological benefits → carsharing usage intention) confirmed H3: Perceived ecological benefits mediate the influence of an environmental claim on carsharing usage intention (*a*_1_*b*_1_= 0.14, 95% *CI* [0.03, 0.29]). In contrast to the postulated effect in H4, a safety claim did not reduce perceived physical risk, as revealed by the direct effect of a safety claim vs. the no-claim condition on perceived physical risk (*a*_4_= 0.16, *p* = *0.55*). However, perceived physical risk had a marginally significant negative direct influence on carsharing usage intention (*b*_2_= −0.09, *p* = 0.09) thereby lending support to H5. The indirect effect of the safety claim condition on carsharing usage intention (safety claim → perceived physical risk → carsharing usage intention) was not significant (*a*_4_*b*_2_= −0.01, 95% *CI* [−0.08, 0.04]); hence, H6 is not confirmed (see Table 2). Figure 3 illustrates the mean difference for the two dependent constructs of perceived ecological benefits and perceived physical risk among the three experimental conditions.

**Table 2:** Relative direct and indirect effects for carsharing usage intention.

	**M**_****1****_ **Perceived ecological benefits**				**M**_****2****_ **Perceived physical risk**				**Y Usage intention**			
**Variable**		**Coeff**.	***SE***	***p***		**Coeff**.	***SE***	***p***		**Coeff**.	***SE***	***p***
Environmental claim	*a_1_*	0.46^*^	0.21	0.03	*a_3_*	0.58^*^	0.27	0.04	*${\text{c}}_{1}^{\prime }$*	0.22	0.22	0.33
Safety claim	*a_2_*	−0.22	0.21	0.29	*a_4_*	0.16	0.27	0.55	$c_{2}^{\prime }$	0.19	0.22	0.38
Perceived ecological benefits (*M_1_*)									*b_1_*	0.30^*^	0.07	0.00
Perceived physical risk (*M_2_*)									*b_2_*	−0.09^+^	0.05	0.09
Familiarity (Cov)	*f_1_*	0.06	0.05	0.22	*g_1_*	0.15^*^	0.06	0.01	*h_1_*	0.15^*^	0.05	0.00
Age (Cov)	*f_2_*	0.00	0.01	0.74	*g_2_*	−0.03^*^	0.01	0.00	*h_2_*	0.00	0.01	0.87
Gender (Cov)	*f_3_*	0.06	0.09	0.50	*g_3_*	0.11	0.12	0.37	*h_3_*	−0.17^+^	0.10	0.07
Education (Cov)	*f_4_*	−0.20	0.16	0.22	*g_4_*	0.14	0.21	0.50	*h_4_*	0.03	0.17	0.88
Advertising appeal (Cov)	*f_5_*	0.12^*^	0.06	0.03	*g_5_*	−0.15^*^	0.07	0.04	*h_5_*	0.29^*^	0.06	0.00
Prior usage (Cov)	*f_6_*	0.27	0.25	0.28	*g_6_*	−0.13	0.32	0.67	*h_6_*	−0.57^*^	0.26	0.03
Constant	*i_*M*1_*	3.51^*^	0.67	0.00	*i_*M*2_*	4.34^*^	0.87	0.00	*i_*y*_*	2.43	0.77	0.00
	***R***^**2**^ **=** **0.11**				***R***^**2**^ **=** **0.13**				***R***^**2**^ **=** **0.30**			
	***F***_**(8, 231)**_ **=** **3.61**, ***p*** **<** **0.01**				***F***_**(8, 231)**_ **=** **4.40**, ***p*** **<** **0.01**				***F***_**(10, 229)**_ **=** **9.59**, ***p*** **<** **0.01**			
											**Coeff**.	**95% CI**
**Relative indirect effects (ecological benefits)**												
Environmental claim										*a_1_b_1_*	0.14^*^	[0.03, 0.29]
Safety claim										*a_2_b_1_*	−0.07	[−0.20, 0.03]
**Relative indirect effects (perceived physical risk)**												
Environmental claim										*a_3_b_2_*	−0.05	[−0.16, 0.01]
Safety claim										*a_4_b_2_*	−0.01	[−0.08, 0.04]

* *Significant at p ≤ 0.05 two tailed*,

+ *Significant at p ≤ 0.10 one tailed, 95% confidence intervals (95% CI)*.

To test the postulated interaction effect of the two experimental conditions claim × information diagnosticity, we considered the two levels of each condition (i.e., environmental claim vs. safety claim × high information diagnosticity vs. low information diagnosticity), but not the control condition (without any label and no diagnosticity at all) in the analysis. A MANCOVA with the two experimental conditions (claim and information diagnosticity) as factor variables and perceived physical risk and perceived ecological benefits as dependent variables as well as the same control variables as in the moderated mediation analysis as covariates reveal a non-significant model [Pillai's trace = 0.00, *F*_(2, 179)_ = 0.08, *p* = 0.92]. Contrary to the expectations specified in H8a, information diagnosticity did not strengthen the influence of an environmental claim on ecological benefits [*F*_(1, 180)_ = 0.00, *p* = 0.96]. Likewise, information diagnosticity did not influence the impact of a safety claim on perceived physical risk [*F*_(1, 180)_ = 0.16, *p* = 0.69]. Hence, H8b is not supported.

## Discussion

The overall objective of this research was to gain a deeper understanding of the opposing underlying mechanisms that drive carsharing usage intention during the Covid-19 pandemic. In this study, we explore two cognitive processes that likely explain carsharing usage intention; namely, the perception of ecological benefits and the perception of physical risk. Results of an online experiment confirm the assumption that perceived ecological benefits caused by environmental claims represent useful means to prompt perceived ecological benefits when using free-floating carsharing, even during the Covid-19 pandemic. Hence, it seems that despite the omnipresence of the Covid-19 pandemic in the media, consumers still pay attention to environmental claims and experience actions against climate change as relevant.

Prior research has already demonstrated the relevance of benefits to explain carsharing usage intention (Acheampong and Siiba, 2020); however, our research is the first that explicitly concentrates on perceived ecological benefits prompted by environmental claims. Given the increased public awareness of CO_2_ emissions and their detrimental effect on climate change, it seems that not only value seeking and convenience benefits (Botsman and Rogers, 2010; Schaefers, 2013) but also environmental benefits represent important drivers of carsharing usage intention. This finding contradicts extant literature that states that ecological benefits are only a nice side effect of using carsharing services (Hartl et al., 2018). One possible explanation for the relevance of ecological benefits in the current study might be the reliance on a label communicating a specific benefit (fewer CO_2_ emissions) of the carsharing service. This finding underpins the relevance of conveying the ecological benefits of a given product or service (Gössling and Buckley, 2016).

Contrary to our expectations, a safety claim did not reduce perceived physical risk in our study. This finding is surprising in light of the emergence of Covid-19 safe labels in various industries. It seems that a Covid-19 safe label is not strong enough to signal the safety aspects of a service. As compared to perceived ecological benefits, consumers might be more sensitive when it comes to risk perceptions. Both effects did not change when providing additional information on specific measures to either protect the environment (environmental claim) or demonstrate how the carsharing provider is able to ensure that the use of the service is Covid-19 safe. Hence, despite the successful manipulation of information diagnosticity, adding more information neither strengthens the positive effect of environmental claims nor made the effect of the safety claim significant. One possible explanation for the absence of a moderating effect of information diagnosticity could be that consumers experience high confidence in making their usage decisions based on the presented label. Prior research confirms that only in the absence of any diagnostic information are consumers sensitive to missing information (Hernandez et al., 2014). However, we can only speculate about the underlying reasons and call for future studies to investigate them in more depth.

Our study confirmed that perceived physical risk is an important negative predictor of carsharing usage intention during the Covid-19 pandemic. Hence, to the best of the authors' knowledge, our research is the first that explores the efficacy of Covid-19 safe labels in the context of free-floating carsharing services. Furthermore, the current study advances perceived risk literature by focusing on an often-neglected perceived risk dimension. Indeed, physical risk has only received research attention in the context of food; however, the Covid-19 pandemic makes consumers experience risk in a new and unobserved way as of yet. Indeed, in the context of carsharing, perceived risk has been researched by relating it to financial risk, performance risk, and social risk of car ownership (Schaefers et al., 2016); however, physical risk of using a carsharing service has not been researched thus far. This seems reasonable since perceived physical risk might not have played such a huge role as compared to financial or performance risk (at least in industrialized countries, where most of these studies were conducted). Nonetheless, with the emergence of the Covid-19 pandemic, health and safety risks have become ubiquitous all over the world and influence consumer purchase decisions to a significant extent in other contexts such as retail shopping too.

Finally, a very interesting finding relates to the positive effect of an environmental claim on perceived risk. In support of our theoretical reasoning, it seems that consumers experience higher levels of perceived physical risk when something other than a safety benefit is communicated during the Covid-19 pandemic. It seems that consumers might draw the wrong conclusion that the carsharing provider cannot meet safety guidelines and hence perceive the risk to be higher. Accordingly, carsharing providers should be cautious with the use of environmental claims during the Covid-19 pandemic. Nevertheless, the only marginal influence of perceived risk on carsharing usage intentions would lead to the practical implication that environmental claims are anyway suitable to motivate users to use carsharing services during the Covid-19 pandemic. Based on the findings of this research, it may nevertheless be a good strategy to rely on other measures to convince consumers of the safety of carsharing services during the pandemic as a safety label is not sufficient to diminish physical risk perceptions. For instance, future research might explore if a safety label certified by third parties might reduce perceived physical risk. Past research reported that labels certified by third parties (e.g., the government) are associated with higher credibility and that a lack of credibility negatively impacts purchase intentions (Teisl et al., 2008; Cai et al., 2017).

The findings yielded in this study also offer important implications for policy makers. First, the insight that eco-labels work even in times of the Covid-19 pandemic and prompt increased carsharing usage might inspire policy makers to establish standardized eco-certification procedures for carsharing providers. Second, since the addition of more information did not enhance the positive effect of eco-claims on perceived environmental benefits or safety-claims on perceived physical risk, our study would suggest that policy makers concentrate their efforts on delivering straightforward information, which can be easily processed. Nevertheless, future research is needed to validate this conclusion. Third, given the negative influence of perceived physical risk on usage intention as revealed in this study, we encourage policy makers to find alternative ways to convince users of the safety of carsharing services. Such an approach might require empirical studies that examine how exactly the coronavirus spreads in shared cars and how this risk could be mitigated. Policy makers might consider funding related studies, similar to a current study exploring the suitability of different devices to understand and quantify air circulation and virus mitigation inside transit buses (Tawfik, 2020). Alternatively, standardized disinfection procedures similar to those developed and monitored by the government might be a promising approach to reduce the risk of a coronavirus infection in shared cars.

As with all empirical studies, our research has some limitations that provide avenues for future research. First, the sample size of 240 respondents, the sampling procedure, and the focus on the German market may limit the generalizability of the results. Future research is required that replicates our findings in other countries, possibly using different sampling strategies. Although the online panel provider Clickworker includes panelists of different educational backgrounds, ages, and genders (as reflected in our sample characteristics), the recruitment of participants for our study is based on a self-selected sample; a type of convenience sample (Colman, 2015). As panelists volunteered to participate in our study, the sample is non-probabilistic: hence, it is not representative of the population under investigation. Future studies might consider collaborating with several carsharing providers, which would offer a larger sample and a random selection of the carsharing population that may be a more representative sample; future studies may also wish to include other stimulus material. In this regard, it has to be noted that other label designs might produce more robust findings or even different findings (e.g., by using another safety claim). Another promising idea for future research would be the conduction of a longitudinal study to explore the long-term effects of the Covid-19 pandemic on carsharing usage. As stated by Cohen (2020b), the Covid-19 pandemic will most likely have a long-lasting impact on cities. The digital transformation in business, work, and commerce challenges the overall purpose of cities (i.e., benefits by concentrating economic activities) and might hamper a recovery of public transport in terms of passenger numbers. To mitigate potential long-lasting negative effects on residents' mobility and/or the climate due to the purchase and use of additional private cars, cities might focus on alternative offerings of modes of transportation, such as publicly-owned carsharing services.

## Data Availability Statement

The raw data supporting the conclusions of this article will be made available by the authors, without undue reservation.

## Ethics Statement

The studies involving human participants were reviewed and approved by Institutional Review Board, Modul University Vienna. The patients/participants provided their written informed consent to participate in this study.

## Author Contributions

MG and CG developed the conceptual research framework, conceived, and designed the experiment. MG was responsible for the data collection and the data analysis. All authors discussed the results, contributed to the final manuscript, critically revised the prior version of the article, and approved the submitted version of this manuscript.

## Conflict of Interest

The authors declare that the research was conducted in the absence of any commercial or financial relationships that could be construed as a potential conflict of interest.

## Footnotes

^1^It has to be noted that public transport was even more negatively affected by the Covid-19 pandemic, with only 20% of individuals returning to German public transport after the lockdown in April (ShareNow, 2020a). To provide a more global perspective, even more dramatic figures are reported in the San Francisco Bay area for public transit: In November, the use of public transportation was 90% below the pre-pandemic level (Cohen, 2020a).

## References

[B1] Acheampong R. A. Siiba A. (2020). Modelling the determinants of car-sharing adoption intentions among young adults: the role of attitude, perceived benefits, travel expectations and socio-demographic factors. Transportation 47, 2557–2580. 10.1007/s11116-019-10029-3

[B2] Akram M. Z. (2020). Inanimate surfaces as potential source of 2019-nCoV spread and their disinfection with biocidal agents. Virusdisease 31, 94–96. 10.1007/s13337-020-00603-0 32656305 PMC7274069

[B3] Andersson L. Gläfke A. Möller T. Schneiderbauer T. (2020). Why Shared Mobility Is Poised to Make a Comeback After the Crisis. Available online at: https://www.mckinsey.com/industries/automotive-and-assembly/our-insights/why-shared-mobility-is-poised-to-make-a-comeback-after-the-crisis (accessed December 16, 2020).

[B4] Andrews J. C. Netemeyer R. G. Burton S. (1998). Consumer generalization of nutrient content claims in advertising. J. Mark. 62, 62–75. 10.1177/002224299806200405

[B5] Atkinson L. Rosenthal S. (2014). Signaling the green sell: the influence of eco-label source, argument specificity, and product involvement on consumer trust. J. Advert. 43, 33–45. 10.1080/00913367.2013.834803

[B6] Bauer R. A. (1960). Consumer behavior as risk taking, in Dynamic Marketing for a Changing World, ed Hancock R. (Chicago: American Marketing Association), 380–398.

[B7] Berry C. Mukherjee A. Burton S. Howlett E. (2015). A COOL effect: the direct and indirect impact of country-of-origin disclosures on purchase intentions for retail food products. J. Retail. 91, 533–542. 10.1016/j.jretai.2015.04.004

[B8] Bert J. Schellong D. Hagenmaier M. Hornstein D. Wegscheider A. K. Palme T. (2020). How Covid-19 Will Shape Urban Mobility. Available online at: https://www.bcg.com/publications/2020/how-covid-19-will-shape-urban-mobility (accessed December 19, 2020).

[B9] Blazquez-Resino J. J. Gutiérrez-Broncano S. Gołab-Andrzejak E. (2020). Neuroeconomy and neuromarketing: the study of the consumer behaviour in the Covid-19 context. Front. Psychol. Available online at: https://www.frontiersin.org/research-topics/14722/neuroeconomy-and-neuromarketing-the-study-of-the-consumer-behaviour-in-the-covid-19-context 10.3389/fpsyg.2022.822856 PMC8965276 35369189

[B10] Botsman R. Rogers R. (2010) What's Mine is Yours: The Rise of Collaborative Consumption. New York, NY: Harper Business.

[B11] Boulding W. Kirmani A. (1993). A consumer-side experimental examination of signaling theory: do consumers perceive warranties as signals of quality? J. Consum. Res. 20, 111–123. 10.1086/209337

[B12] Cai Z. Xie Y. Aguilar F. X. (2017). Eco-label credibility and retailer effects on green product purchasing intentions. For. Policy. Econ. 80, 200–208. 10.1016/j.forpol.2017.04.001

[B13] Clickworker (2021). What Does A Clickworker Do? Available online at: https://www.clickworker.com/clickworker-job/#:~:text=clickworker%20provides%20digital%20tasks%20to,personal%20computer%20with%20Internet%20access.&text=Register%20as%20a%20Clickworker (accessed January 8, 2021).

[B14] Codagnone C. Veltri G. A. Bogliacino F. Lupiáñez-Villanueva F. Gaskell G. Ivchenko A.. (2016). Labels as nudges? An experimental study of car eco-labels. Econ. Polit. 33, 403–432. 10.1007/s40888-016-0042-2

[B15] Cohen A. (2020a). Considerations for Social Distancing on Public Transportation During the Covid-19 Recovery. Available online at: https://transweb.sjsu.edu/sites/default/files/2065-Cohen-Social-Distancing-Public-Transit.pdf (accessed January 09, 2021).

[B16] Cohen A. (2020b). COVID-19's Potential Impact on Cities: Five Trends and Indicators to Watch. Available online at: https://transweb.sjsu.edu/research/2062-Risk-Urban-Decline (accessed January 09, 2021).

[B17] Colman A. A. (2015) A Dictionary of Psychology, 4th Edn. Oxford: Oxford University Press.

[B18] Deloitte Germany (2017). Carsharing in Europe. Available online at: https://www2.deloitte.com/content/dam/Deloitte/de/Documents/consumer-industrial-products/CIP-Automotive-Car-Sharing-in-Europe.pdf (accessed December 10, 2020).

[B19] Deloitte Italy (2020). From Now on: Mobility Boost, a New Phase Coming. Available online at: https://www2.deloitte.com/content/dam/Deloitte/it/Documents/strategy/Deloitte_Future_of_mobility_COVID19_ENG.pdf (accessed December 19, 2020).

[B20] Douthitt R. (1995). Consumer risk perception and recombinant bovine growth hormone: the case for labelling dairy products made from untreated herd milk. J. Public Policy Mark. 14, 328–332. 10.1177/074391569501400215

[B21] Efthymiou D. Antoniou C. Waddell P. (2013). Factors affecting the adoption of vehicle sharing systems by young drivers. Transp. Policy 29, 64–73. 10.1016/j.tranpol.2013.04.009

[B22] El Keshky M. E. Basyouni S. S. Al Sabban A. M. (2020). Getting through covid-19: the pandemic's impact on the psychology of sustainability, quality of life, and the global economy–a systematic review. Front. Psychol. 11:585897. 10.3389/fpsyg.2020.585897 33281683 PMC7688781

[B23] Field A. P. (2009). Discovering Statistics Using SPSS: (and Sex and Drugs and Rock ‘n’ Roll). London: SAGE.

[B24] Fiorillo L. Cervino G. Matarese M. D'Amico C. Surace G. Paduano V.. (2020). COVID-19 surface persistence: a recent data summary and its importance for medical and dental settings. Int. J. Environ. Res. Public Health. 17:3132. 10.3390/ijerph17093132 32365891 PMC7246498

[B25] Firnkorn J. (2012). Triangulation of two methods measuring the impacts of a free-floating carsharing system in Germany. Transp. Res. A 46, 1654–1672. 10.1016/j.tra.2012.08.003

[B26] Firnkorn J. Müller M. (2015). Free-floating electric carsharing-fleets in smart cities: the dawning of a post-private car era in urban environments? Environ Sci Policy 45, 30–40. 10.1016/j.envsci.2014.09.005

[B27] Gates B. (2020). COVID-19 is Awful. Climate Change Could be Worse. Available online at: https://www.gatesnotes.com/Energy/Climate-and-COVID-19 (accessed December 20, 2020).

[B28] German Aerospace Center (2020a). DLR-Befragung: Wie Verändert Corona Unsere Mobilität? [DLR Survey: How is Corona Changing our Mobility?]. Available online at: https://www.dlr.de/content/de/artikel/news/2020/02/20200506_dlr-befragung-wie-veraendert-corona-unsere-mobilitaet.html (accessed January 9, 2021).

[B29] German Aerospace Center (2020b). DLR-Befragung: Zweite DLR-Studie zu Corona und Mobilität: Öffentliche verlieren, Individualverkehr gewinnt an Bedeutung [Second DLR Study on Corona and Mobility: Public Transport is Losing, Private Transport is Gaining in Importance]. Available online at: https://www.dlr.de/content/de/artikel/news/2020/03/20200928_zweite-dlr-studie-zu-corona-und-mobilitaet-oeffentliche-verlieren.html (accessed January 9, 2021).

[B30] German Aerospace Center (2020c). Zweite DLR-Befragung: Wie Verändert Corona Unsere Mobilität? [Second DLR Survey: How is Corona Changing our Mobility?]. Available online at: https://verkehrsforschung.dlr.de/de/news/zweite-dlr-befragung-wie-veraendert-corona-unsere-mobilitaet (accessed January 9, 2021).

[B31] Goldman E. (2020). Exaggerated risk of transmission of COVID-19 by fomites. Lancet Infect Dis. 20, 892–893. 10.1016/S1473-3099(20)30561-2 32628907 PMC7333993

[B32] Google (2020) Google Covid-19 Community Mobility Report. Available online at: https://www.google.com/covid19/mobility/ (accessed December 27, 2020).

[B33] Gössling S. Buckley R. (2016). Carbon labels in tourism: persuasive communication? J. Clean. Prod. 111, 358–369. 10.1016/j.jclepro.2014.08.067

[B34] Gutierrez A. M. Chiu A. S. Seva R. (2020). A proposed framework on the affective design of eco-product labels. Sustainability 12:3234. 10.3390/su12083234

[B35] Hartl B. Sabitzer T. Hofmann E. Penz E. (2018). “Sustainability is a nice bonus”: the role of sustainability in carsharing from a consumer perspective. J. Clean. Prod. 202, 88–100. 10.1016/j.jclepro.2018.08.138

[B36] Hartmann P. Apaolaza-Ibáñez V. (2012) Consumer attitude purchase intention toward green energy brands: the roles of psychological benefits environmental concern. J. Bus. Res. 65, 1254–1263. 10.1016/j.jbusres.2011.11.001

[B37] Hawksworth J. Vaughan R. (2014). The Sharing Economy–Sizing the Revenue Opportunity. Available online at: https://pwc.blogs.com/files/sharing-economy-final_0814.pdf (accessed December 19, 2020).

[B38] Hayes A. F. (2017). Introduction to Mediation, Moderation, and Conditional Process Analysis: A Regression-Based Approach. 2nd Edn. New York, NY: Guilford Press.

[B39] Hernandez J. M. Han X. Kardes F. R. (2014). Effects of the perceived diagnosticity of presented attribute and brand name information on sensitivity to missing information. J. Bus. Res. 67, 874–881. 10.1016/j.jbusres.2013.07.006

[B40] HRS (2020) Clean & Safe Protocol. Available online at: https://www.hrs.com/corporate/solutions/clean-and-safe-protocol/ (accessed December 12 2020).

[B41] Ikonen I. Sotgiu F. Aydinli A. Verlegh P. W. (2020). Consumer effects of front-of-package nutrition labeling: an interdisciplinary meta-analysis. J. Acad. Mark. Sci. 48, 360–383. 10.1007/s11747-019-00663-9

[B42] Jacoby J. Kaplan L. B. (1972). The components of perceived risk, in SV - Proceedings of the Third Annual Conference of the Association for Consumer Research, ed Venkatesan M. (Chicago, IL: Association for Consumer Research), 382–393.

[B43] Jochem P. Frankenhauser D. Ewald L. Ensslen A. Fromm H. (2020). Does free-floating carsharing reduce private vehicle ownership? The case of SHARE NOW in European cities. Transp. Res. A 141, 373–395. 10.1016/j.tra.2020.09.016 33052178 PMC7544607

[B44] Kopp J. Gerike R. Axhausen K. W. (2015). Do sharing people behave differently? An empirical evaluation of the distinctive mobility patterns of free-floating car-sharing members. Transportation 42, 449–469. 10.1007/s11116-015-9606-1

[B45] Kortum K. Schönduwe R. Stolte B. Bock B. (2016). Free-floating carsharing: city-specific growth rates and success factors. Transp. Res. Procedia 19, 328–340. 10.1016/j.trpro.2016.12.092

[B46] Larceneux F. Benoit-Moreau F. Renaudin V. (2012). Why might organic labels fail to influence consumer choices? Marginal labelling and brand equity effects. J. Consum. Policy 35, 85–104. 10.1007/s10603-011-9186-1

[B47] Lindloff K. Pieper N. Bandelow N. Woisetschläger W. (2014) Drivers of carsharing diffusion in Germany: an actor-centered approach. Int. J. Automot. Technol. 14, 217–245. 10.1504/IJATM.2014.065291

[B48] Loomba R. S. Aggarwal G. Aggarwal S. Flores S. Villarreal E. G. Farias J. S.. (2021). Disparities in case frequency and mortality of coronavirus disease 2019 (COVID-19) among various states in the United States. Ann. Med. 53, 151–159. 10.1080/07853890.2020.1840620 33138653 PMC7877922

[B49] Loureiro M. L. Lotade J. (2005). Do fair trade and eco-labels in coffee wake up the consumer conscience? Ecol. Econ. 53, 129–138. 10.1016/j.ecolecon.2004.11.002

[B50] Lyons K. (2019). Climate Crisis Already Causing Deaths and Childhood Stunting, Report Reveals. Available online at: https://www.theguardian.com/environment/2019/jul/31/climate-crisis-already-causing-deaths-and-childhood-stunting-report-reveals (accessed December 10, 2020).

[B51] Martin E. Shaheen S. (2016). Impacts of car2go on Vehicle Ownership, Modal Shift, Vehicle Miles Traveled, and Greenhouse Gas Emissions: An Analysis of Five North American Cities. Working Paper. Available online at: http://innovativemobility.org/wp-content/uploads/2016/07/Impactsofcar2go_FiveCities_2016.pdf (accessed January 09, 2021).

[B52] Matzler K. Veider V. Kathan W. (2015). Adapting to the sharing economy. MIT Sloan Manag. Rev. 56, 71–77. Available online at: https://sloanreview.mit.edu/article/adapting-to-the-sharing-economy/ (accessed January 9, 2021).

[B53] Menon G. Raghubir P. Schwarz N. (1995). Behavioral frequency judgments: an accessibility-diagnosticity framework. J. Consum. Res. 22, 212–228. 10.1086/209446

[B54] Mishra D. P. Heide J. B. Cort S. G. (1998) Information asymmetry levels of agency relationships. J. Mark. Res. 35, 277–295. 10.1177/002224379803500301

[B55] Mitra K. Reiss M. C. Capella L. M. (1999). An examination of perceived risk, information search and behavioral intentions in search, experience and credence services. J. Serv. Mark. 13, 208–220. 10.1108/08876049910273763

[B56] Mohd S. N. (2016). Green product purchase intention: impact of green brands, attitude, and knowledge. Br. Food J. 118, 2893–2910. 10.1108/BFJ-06-2016-0295

[B57] Münzel K. Piscielli L. Boon W. Frenken K. (2019). Different business models – different users? Uncovering the motives and characteristics of business-to-consumer and peer-to-peer carsharing adopters in the Netherlands. Transport Res. D Transport. Environ. 73, 276–306. 10.1016/j.trd.2019.07.001

[B58] Muro-Rodríguez A. I. Perez-Jiménez I. R. Gutiérrez-Broncano S. (2017). Consumer behavior in the choice of mode of transport: a case study in the toledo-madrid corridor. Front. Psychol. 8:1011. 10.3389/fpsyg.2017.01011 28676776 PMC5476976

[B59] Nijland H. van Meerkerk J. (2017). Mobility and environmental impacts of car sharing in the Netherlands. Environ. Innov. Soc. Transit. 23, 84–91. 10.1016/j.eist.2017.02.001

[B60] Nourinejad M. Roorda M. J. (2015). Carsharing operations policies: a comparison between one-way and two-way systems. Transportation 42, 497–518. 10.1007/s11116-015-9604-3

[B61] Orazi D. C. Chan E. Y. (2018). They did not walk the green talk!: how information specificity influences consumer evaluations of disconfirmed environmental claims. J. Bus. Ethics 163, 107–123. 10.1007/s10551-018-4028-6

[B62] Park J. Lennon S. J. Stoel L. (2005). On-line product presentation: effects on mood, perceived risk, and purchase intention. Psychol. Mark. 22, 695–719. 10.1002/mar.20080

[B63] Pek J. Wong O. Wong A. C. M. (2018) How to address non-normality: taxonomy of approaches, reviewed illustrated. Front. Psychol. 9:2104. 10.3389/fpsyg.2018.02104 30459683 PMC6232275

[B64] Ramos E. M. S. Bergstad C. J. Chicco A. Diana M. (2020). Mobility styles and carsharing use in Europe: attitudes, behaviours, motives and sustainability. Eur. Transp. Res. Rev. 12, 1–12. 10.1186/s12544-020-0402-4

[B65] Schaefers T. (2013). Exploring carsharing usage motives: a hierarchical means-end chain analysis. Transport Res. A Policy Pract. 47, 69–77. 10.1016/j.tra.2012.10.024

[B66] Schaefers T. Lawson S. J. Kukar-Kinney M. (2016). How the burdens of ownership promote consumer usage of access-based services. Mark Lett. 27, 569–577. 10.1007/s11002-015-9366-x

[B67] Shaheen S. Cohen A. Broader J. Davis R. Brown L. Neelakantan R. (2020). Mobility on Demand Planning and Implementation: Current Practices, Innovations, and Emerging Mobility Futures. Available online at: https://rosap.ntl.bts.gov/view/dot/50553 (accessed January 09, 2021)

[B68] Shaheen S. A. Chan N. D. Micheaux H. (2015). One-way carsharing's evolution and operator perspectives from the Americas. Transportation 42, 519–536. 10.1007/s11116-015-9607-0

[B69] ShareNow (2020a). Fakten und Zahlen: Carsharing Während COVID-19. Available online at: https://www.share-now.com/at/de/corona-carsharing-facts-figures/ (accessed January 9, 2021).

[B70] ShareNow (2020b). A New Era of Car-Sharing. Available online at: https://www.share-now.com/ (accessed December 29, 2020).

[B71] Simonin B. L. Ruth J. A. (1998). Is a company known by the company it keeps? Assessing the spillover effects of brand alliances on consumer brand attitudes. J. Mark. Res. 35, 30–42. 10.1177/002224379803500105

[B72] Spence M. (1973). Job market signaling. Q. J. Econ. 87, 355–374. 10.2307/1882010

[B73] Stone R. N. Grønhaug K. (1993). Perceived risk: further considerations for the marketing discipline. Eur. J. Mark. 27, 39–50. 10.1108/03090569310026637

[B74] Suman R. Javaid M. Haleem A. Vaishya R. Bahl S. Nandan D. (2020). Sustainability of coronavirus on different surfaces. J. Clin. Exp. Hepatol. 10, 386–390. 10.1016/j.jceh.2020.04.020 32377058 PMC7201236

[B75] Tabachnick B. G. Fidell L. S. (2000). Using Multivariate Statistics. Boston, MA: Allyn and Bacon.

[B76] Tawfik A. (2020). COVID-19 Transit Bus Air Circulation and Virus Mitigation Study. Available online at: https://transweb.sjsu.edu/csutc/research/utc/COVID-19-Transit-Bus-Air-Circulation-and-Virus-Mitigation-Study (accessed January 09, 2021)

[B77] Taylor J. W. (1974). The role of risk in consumer behavior: a comprehensive and operational theory of risk taking in consumer behavior. J. Mark. 38, 54–60. 10.1177/002224297403800211

[B78] Teisl M. F. Rubin J. Noblet C. L. (2008). Non-dirty dancing? Interactions between eco-labels and consumers. J. Econ. Psychol. 29, 140–59. 10.1016/j.joep.2007.04.002

[B79] Terrien C. Maniak R. Chen B. Shaheen S. (2016). Good practices for advancing urban mobility innovation: a case study of one-way carsharing. Res. Transport. Bus. Manage. 20, 20–32. 10.1016/j.rtbm.2016.08.001

[B80] Thakur R. Srivastava M. (2014). Adoption readiness, personal innovativeness, perceived risk and usage intention across customer groups for mobile payment services in India. Internet Res. 24, 369–392. 10.1108/IntR-12-2012-0244

[B81] Transport Canada (2020). Federal Safety Guidance to Protect Drivers and Limit the Spread of COVID-19 in Commercial Vehicle Operations. Available online at: https://tc.canada.ca/en/initiatives/covid-19-measures-updates-guidance-issued-transport-canada/federal-safety-guidance-protect-drivers-limit-spread-covid-19-commercial-vehicle-operations (accessed January 09, 2021)

[B82] Van Doremalen N. Bushmaker T. Morris D. H. Holbrook M. G. Gamble A. Williamson B. N.. (2020). Aerosol and surface stability of SARS-CoV-2 as compared with SARS-CoV-1. N. Engl. J. Med. 382, 1564–1567. 10.1056/NEJMc2004973 32182409 PMC7121658

[B83] Vaskelainen T. Münzel K. (2018). The effect of institutional logics on business model development in the sharing economy: the case of German carsharing services. Acad. Manag. Discov. 4, 279–293. 10.5465/amd.2016.0149

[B84] Venkatesh V. Thong Y. L. Xu X. (2012). Consumer acceptance and use of information technology: extending the unified theory of acceptance and use of technology. MIS Qu. 36, 157–178. 10.2307/41410412

[B85] Visit Brussels (2020). The Label Focusing on Health Safety for the Brussels Tourism Sector. Available online at: https://visit.brussels/en/article/the-label-focussing-on-health-safety-for-the-brussels-tourism-sector (accessed December 12, 2020).

[B86] Wappelhorst S. Sauer M. Hinkeldein D. Bocherding A. Glaß T. (2014). Potential of electric carsharing in urban and rural areas. Transport. Res. Procedia 4, 374–386. 10.1016/j.trpro.2014.11.028

[B87] Wikipedia Commons (2014). File:BMW i3 – Frontansicht, 5. Oktober 2014, Düsseldorf.jpg - Wikimedia Commons. Available online at: https://commons.wikimedia.org/wiki/File:BMW_i3_%E2%80%93_Frontansicht_5._Oktober_2014,_D%C3%BCsseldorf.jpg (accessed December 20, 2020).

[B88] Worland J. (2020). Climate Change Could Cause More Annual Deaths Than Infectious Disease by 2100. Available online at: https://time.com/5876229/climate-change-death-rate/ (accessed December 19, 2020).

[B89] Yeung R. Yee W. Morris J. (2010). The effects of risk-reducing strategies on consumer perceived risk and on purchase likelihood. Br. Food J. 112, 306–322. 10.1108/00070701011029174

